# Evidence for direct interaction between the oncogenic proteins E6 and E7 of high-risk human papillomavirus (HPV)

**DOI:** 10.1016/j.jbc.2023.104954

**Published:** 2023-06-23

**Authors:** JiaWen Lim, Hauke Lilie, Hubert Kalbacher, Nora Roos, Desiree Isabella Frecot, Maximilian Feige, Marcel Conrady, Tobias Votteler, Alexandra Cousido-Siah, Giada Corradini Bartoli, Thomas Iftner, Gilles Trave, Claudia Simon

**Affiliations:** 1 Institute of Medical Virology and Epidemiology of Viral Diseases, University Hospital Tuebingen, Tuebingen, Germany; 2 Institute of Biochemistry and Biotechnology, Martin-Luther-University Halle-Wittemberg, Halle-Wittemberg, Germany; 3 Interfaculty Institute of Biochemistry, Eberhard-Karls-University Tuebingen, Tuebingen, Germany; 4 Equipe Labellisée Ligue 2015, Department of Integrative Biology, Institut de Génétique et de Biologie Moléculaire et Cellulaire, CNRS, INSERM, UdS, Illkirch, France

**Keywords:** human papillomavirus, high-risk, intraviral protein–protein interaction, E6 carcinogenic protein, E7 carcinogenic protein

## Abstract

Human papillomaviruses (HPVs) are DNA tumor viruses that infect mucosal and cutaneous epithelial cells of more than 20 vertebrates. High-risk HPV causes about 5% of human cancers worldwide, and the viral proteins E6 and E7 promote carcinogenesis by interacting with tumor suppressors and interfering with many cellular pathways. As a consequence, they immortalize cells more efficiently in concert than individually. So far, the networks of E6 and E7 with their respective cellular targets have been studied extensively but independently. However, we hypothesized that E6 and E7 might also interact directly with each other in a novel interaction affecting HPV-related carcinogenesis. Here, we report a direct interaction between E6 and E7 proteins from carcinogenic HPV types 16 and 31. We demonstrated this interaction *via* cellular assays using two orthogonal methods: coimmunoprecipitation and flow cytometry–based FRET assays. Analytical ultracentrifugation of the recombinant proteins revealed that the stoichiometry of the E6/E7 complex involves two E7 molecules and two E6 molecules. In addition, fluorescence polarization showed that (I) E6 binds to E7 with a similar affinity for HPV16 and HPV31 (in the same micromolar range) and (II) that the binding interface involves the unstructured N-terminal region of E7. The direct interaction of these highly conserved papillomaviral oncoproteins may provide a new perspective for studying HPV-associated carcinogenesis and the overall viral life cycle.

To date, there are more than 200 types of human papillomaviruses (HPVs) known, which have been classified into five genera (alpha, beta, gamma, mu, and nu) based on their L1 nucleotide sequences (1). HPVs from the alpha genus are further divided into high risk and low risk by the International Agency for Research on Cancer based on their potential carcinogenic properties (2). High-risk HPV (HPV 16, 18, 31, 33, 35, 39, 45, 51, 52, 56, 58, 59, 66, and 68) causes approximately 5% of cancers worldwide, with HPV16 being the most carcinogenic (3, 4). The viral proteins E6 and E7 are crucial in targeting many cellular proteins and a wide range of cellular processes to develop and maintain carcinogenesis, as reviewed (5, 6).

E7 proteins are highly conserved. E7 consists of three conserved regions, namely CR1, CR2, and CR3 (7). CR1 and CR2 are highly acidic and presumably disordered (8), whereas CR3 consists of two CxxC zinc-binding motifs (9, 10). CR1 and CR2 play critical roles in cellular transformation and immortalization, with CR2 exhibiting an LxCxE motif, the dominant binding site for the retinoblastoma protein (pRb) (11, 12). CR3 of E7 triggers the formation of stable dimers (9, 13, 14), and it binds protein tyrosine phosphatase nonreceptor type 14 (PTPN14) (15). Most E7 proteins target the two tumor suppressors pRb and PTPN14 for proteasomal degradation *via* the recruitment of cullin 2 and UBR4 ubiquitin ligase, respectively, leading to uncontrolled cell cycle progression and mediating carcinogenesis (15, 16, 17, 18, 19, 20). An elevation of the p53 expression level in the presence of E7 proteins that could lead to apoptosis has been reported previously (21). However, this is overcome by the expression of the E6 protein.

E6 is less conserved among papillomaviruses as compared with E7. However, all E6 consists of four CxxC zinc-binding motifs forming two domains, the E6N and E6C (22, 23). It is known that E6 targets LxxLL motifs of several cellular proteins with affinities in the micromolar range and binds the LxxLL motif at the cleft between E6N and E6C (22, 23). The most extensively studied model is the recruitment of E3 ubiquitin ligase E6–associated protein (E6AP) by E6; the complex binds tumor suppressor p53 resulting in ubiquitination and degradation of p53 (22, 23, 24). This, in turn, interferes with p53-dependent apoptosis and cell cycle arrest (25). In addition, a unique PDZ-binding domain found only at the C terminus of E6 from high-risk alpha HPV types allows these E6s to target PDZ-containing proteins such as DLG-1 and MAGI-1, dysregulating the cellular polarity (26, 27, 28, 29).

E6 and E7 cooperate to drive cellular transformation and immortalization of human keratinocytes (30, 31, 32). This was observed with the indefinite growth of keratinocytes in the presence of both E6 and E7, whereas E6 alone does not immortalize human keratinocytes (30). A direct interaction between E6 and E7 has not been described so far. Here, we demonstrate an interaction between E6 and E7 proteins of several HPV types in flow cytometry–based FRET assays (fluorescence-activated cell sorting [FACS]–FRET) and *in vitro* using analytical ultracentrifugation and fluorescence polarization (FP).

## Results

### Evidence supporting interaction between E6 and E7 in cellular assays

**Figure 1:**
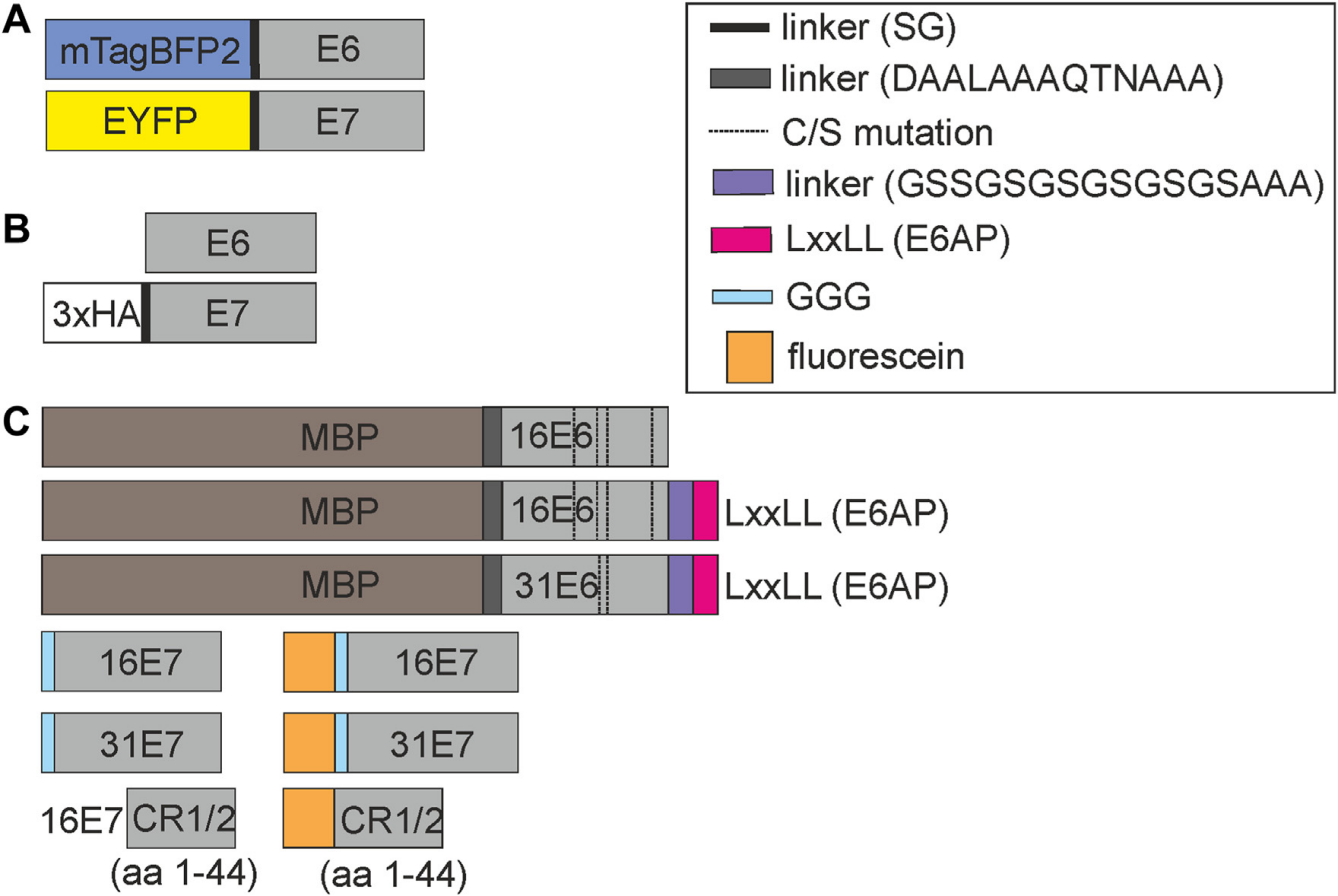
**HPV E6 and E7 constructs used in the respective experiments.***A*, the E6 and E7 of HPV 16, 18, 31, 8, 38 used in the FACS–FRET assay were N-terminally fused with mTagBFP2 and EYFP, respectively. *B*, the untagged E6 and the N-terminally 3xHA-tagged E7 constructs of HPV 16 and HPV 31 were used in coimmunoprecipitation. *C*, the various constructs of HPV 16 and HPV 31 were used to produce purified recombinant proteins for analytical ultracentrifugation and fluorescence polarization. EYFP, enhanced YFP; FACS, fluorescence-activated cell sorting; HA, hemagglutinin; HPV, human papillomavirus.

**Figure 2:**
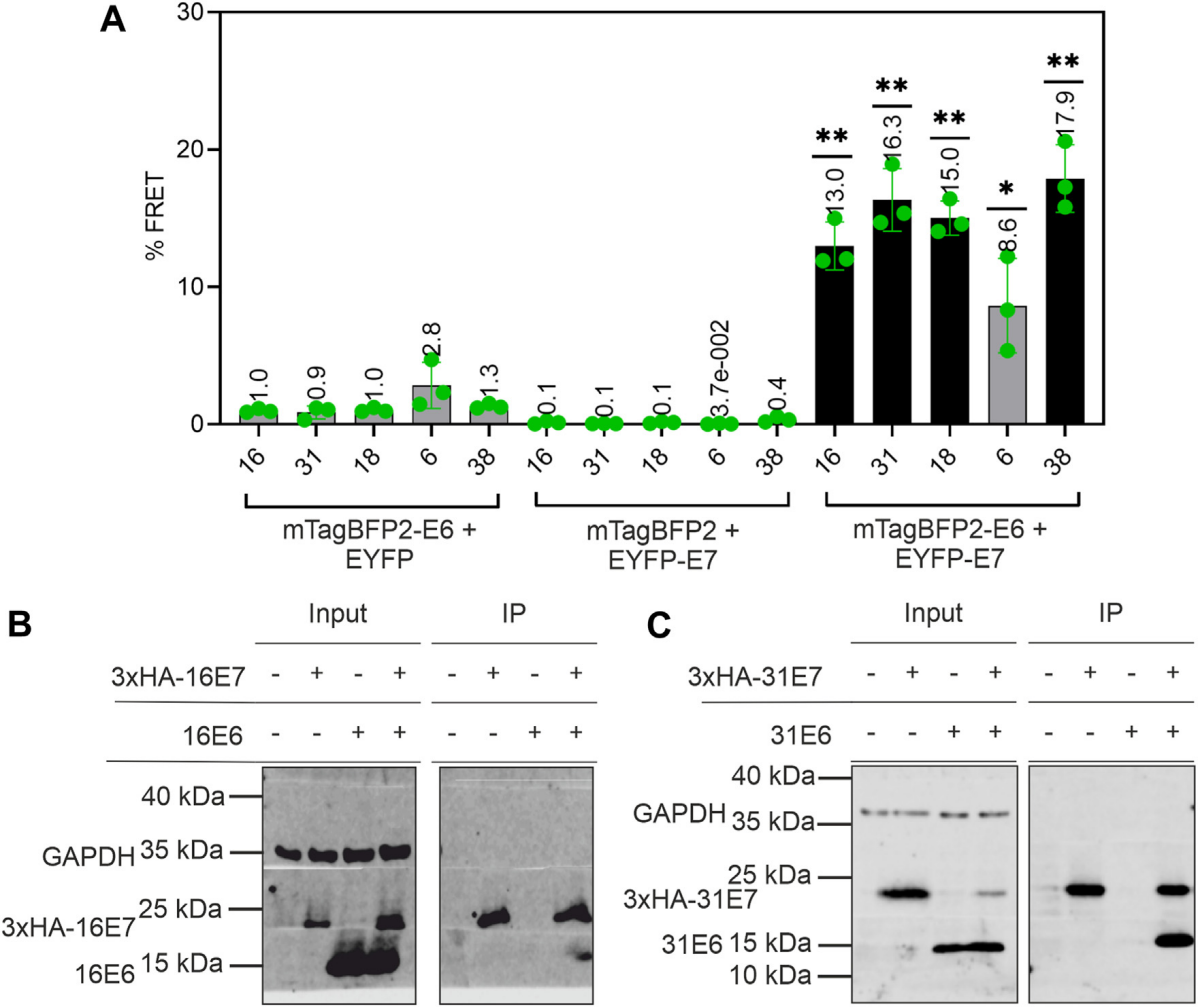
**E6 interacts with E7 in cell-based assays.***A*, C33A coexpressing mTagBFP2-E6 and EYFP-E7 of each HPV type were subjected to FACS–FRET and revealed a positive FACS–FRET signal for E6 and E7 proteins from high-risk alpha HPV16, HPV31, HPV18, and beta HPV38, indicating an interaction. The signal for HPV6 is below the threshold; thus, the interaction is unclear. Data are derived from the mean value of three independent biological replicates. The error bars are plotted to represent the standard deviation of the mean value from the three independent biological replicates. The *green dots* represent the scatter dot plot of the three independent biological replicates. *p* Value was calculated with one-sample *t* test, where ∗∗ = *p* > 0.005 and ∗ = *p* > 0.01. Please see Supporting information SI2 for detailed statistical data (Table S2*A*) and the number of FRET-positive cells (Fig. S2 and Table S2*B*). *B* and *C*, 70 μg cell lysates from C33A cells (*input*) or 25 μl of proteins precipitated with α-HA antibody (IP) were subjected to immunoblot analysis. The membrane was cut at respective marker bands (above 40 kDa, below 35 kDa, and above 15 kDa) before probing with respective antibodies. Later, the membrane strips were aligned and visualized at the same time at LI-COR Odyssey Fc. The untagged E6 of alpha high-risk HPV16 (*B*) or HPV31 (*C*) was coimmunoprecipitated with 3xHA-16E7 or 3xHA-31E7, respectively. Please see Fig. S4 in SI4 for the full blot. EYFP, enhanced YFP; FACS, fluorescence-activated cell sorting; HPV, human papillomavirus; IP, immunoprecipitation.

To screen the interactions between E6 and E7 *via* FACS–FRET, C33A cells were cotransfected with plasmids encoding mTagBFP2-E6 and enhanced YFP (EYFP)-E7 (Fig. 1*A*) of the same HPV type for all HPV types tested. Furthermore, we verified the interaction of E6 and E7 from two high-risk HPVs, 16 and 31, by coimmunoprecipitation (co-IP). A percent FRET signal of at least 10% and at least 500 FRET-positive cells indicates an interaction. Negative controls (EYFP + mTagBFP2-E6, mTagBFP2 + EYFP-E7) and positive control (fusion of mTagBFP2-EYFP) were always included. Most of these controls showed less than 1.0% FACS–FRET signal for each HPV tested (Fig. 2*A* and Table S2*A*), indicating no binding event. The mTagBFP2-6E6 and mTagBFP2-38E6 coexpressed with EYFP show percent FRET signal of more than 1.0%. However, this signal could be neglected because of the low number of less than 50 FRET-positive cells (Fig. S2 and Table S2*B*). Finally, positive FRET signals were observed for alpha high-risk HPV16 (13.0 ± 1.7%), HPV31 (16.3 ± 2.3%), HPV18 (15.0 ± 1.2%), and beta HPV38 (17.9 ± 2.5%) with more than 750 FRET-positive cells (Fig. S2 and Table S2*B*). Because of the different expression levels of mTagBFP2-E6 and EYFP-E7 (Fig. S3 and Table S3, *A* and *B*), only qualitative evaluation could be applied. Hence, comparing the signals of various FRET pairs quantitatively should be avoided. Notably, the expression of HPV6 E6 proteins was extremely low (Fig. S3 and Table S3, *A* and *B*), leading to a low percent FRET of 8.6 ± 3.4% (Fig. 2*A*) and low FRET-positive cells of 126 cells (Fig. S2), below the threshold applied for the analysis.

Next, we performed hemagglutinin (HA) co-IP with 3XHA-E7 and E6 (Figs. 1*B* and S4) proteins coexpressed in C33A *via* plasmid DNA transfection to validate the result of the FACS–FRET. It was seen that the HPV16 E6 proteins bind nonspecifically to the HA magnetic beads but not HPV31 E6 proteins (data not shown). Hence, for HPV16, we employed a 3xHA peptide to conduct native elution of the complex to eliminate the nonspecific-bound 16E6 protein in the coelution as described (33). Figure 2, *B* and *C* showed that both untagged HPV16 E6 and HPV31 E6 bind to 3xHA-tagged HPV16 E7 and HPV31 E7, respectively.

In summary, the data suggest that the E6 and E7 proteins of carcinogenic HPV16 and HPV31 formed a complex in cell-based assays. The interaction was not only observed for high-risk alpha HPV16 and HPV31 but also for high-risk alpha HPV18 and beta HPV38 through FACS–FRET.

### Two E7 molecules recruit two E6 molecules according to analytical ultracentrifugation

To understand the stoichiometry of the complex, we carried out analytical ultracentrifugation (AUC) with purified recombinant E6 and E7 proteins. Two measurements were performed with a complex formed at a 1:1 molar ratio of monomers, including sedimentation velocity and sedimentation equilibrium. Measuring E7 at 280 nm at a lower concentration is challenging because of the low extinction coefficient. Hence, we labeled 16E7 with fluorescein dye *via sortase A* labeling technique as described in Supporting information S7 and measured the labeled E7 (fl-16E7) signal at 495 nm in the presence and absence of maltose-binding protein (MBP)-16E6_4C4S-LxxLL.

**Figure 3:**
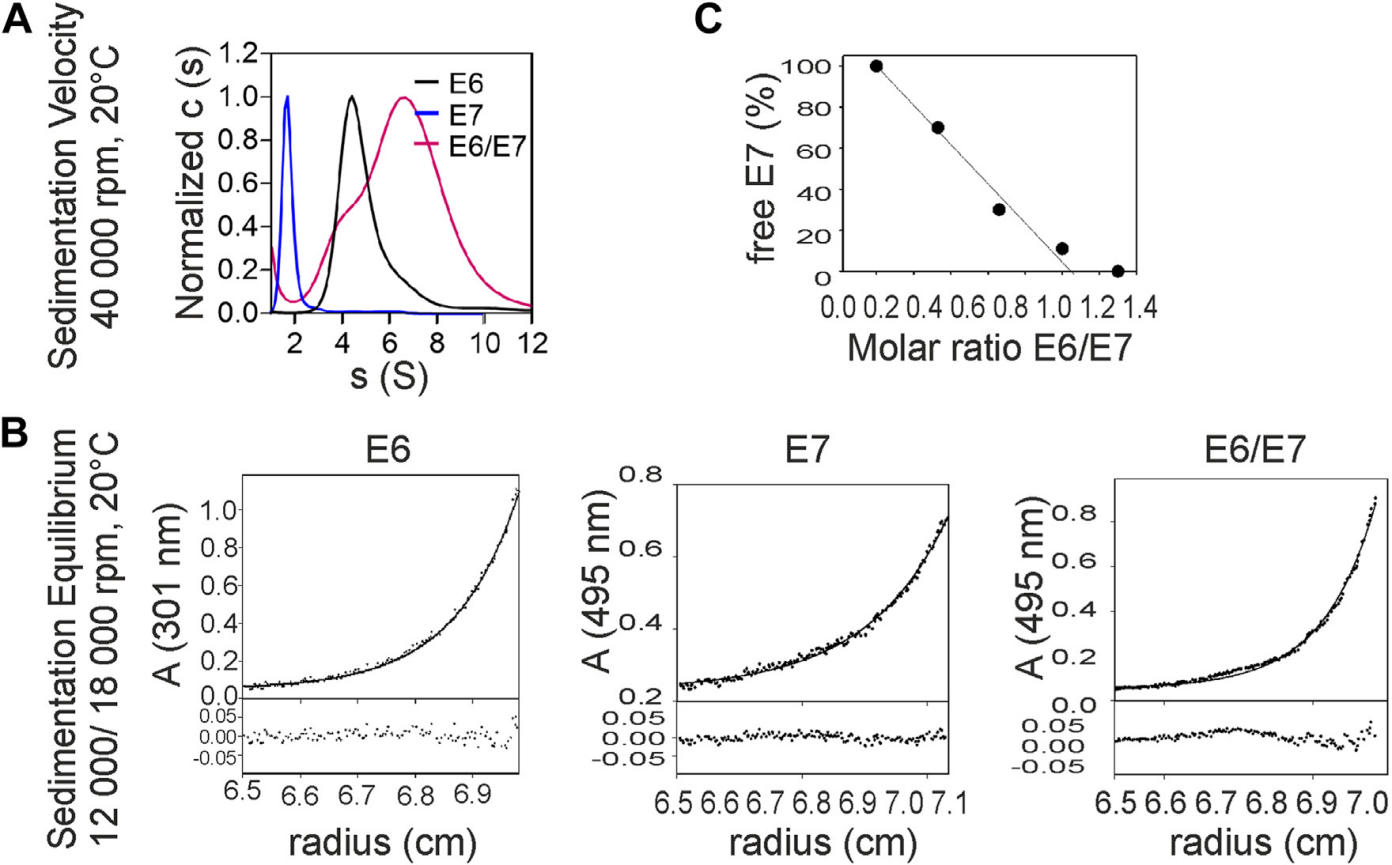
**Stoichiometry of E6–E7 complex.***A*, the sedimentation velocity revealed the sedimentation coefficient of MBP-16E6_4C4S-LxxLL (*black*, named E6), fl-16E7 (*blue*, named E7), and the 1:1 molar ratio mixture of E6 and E7 (*magenta*, named E6/E7) calculated with SEDFIT, version 12.52. E6 was monitored at 280 nm, whereas the E7 and E6/E7 were monitored at 495 nm. *B*, the sedimentation equilibrium of MBP16E6_4C4S-LxxLL (named E6), fl-16E7 (named E7), and the 1:1 molar ratio mixture of E6 and E7 (named E6/E7). *C*, the amount of free fl-16E7 (named E7) decreased with an increasing amount of MBP16E6_4C4S-LxxLL, and it was not detected at a molar ratio of approximately 1:1. MBP, maltose-binding protein.

The sedimentation velocity measurement showed a shift in the sedimentation profile for the complex compared with MBP-16E6_4C4S-LxxLL and fl-16E7 alone (Fig. S6). It revealed a sedimentation coefficient (Fig. 3*A*) for two species of s_app_ = 6.70 (major species, ∼89%) and s_app_ = approximately 4.0 (minor species, ∼11%). The sedimentation coefficients of MBP-16E6_4C4S-LxxLL and fl-16E7 alone were s = 4.30 and s = 1.70, respectively. The shift of the sedimentation coefficient from 1.70 and 4.30 to ∼6.70 indicated the complex formation of MBP-16E6_4C4S-LxxLL and fl-16E7. In addition, the formation of a clear sedimenting species neglected the possible formation of heterogeneous agglomerates, which the high density of cysteines in both proteins may cause. Furthermore, the minor species seen in the complex with s = ∼4.0 may be the intermediate species of the complex, as this species was monitored at 495 nm for the signal from fl-16E7.

For further characterization, we determined the molecular weight (MW) of MBP-16E6_4C4S-LxxLL of 63.4 ± 4.9 kDa, fl-16E7 of 19.7 ± 2.1 kDa, and MBP-16E6_4C4S-LxxLL/fl-16E7 of 142.0 ± 6.5 kDa with sedimentation equilibrium runs (Fig. 3*B*). These MWs fitted the theoretical MWs of the MBP-16E6_4C4S-LxxLL monomer and fl-16E7 dimer alone (Table 1). The MW_app_ of the complex could correspond to 2× MBP-16E6_4C4S-LxxLL and 2× fl-16E7. We further titrated MBP-16E6_4C4S-LxxLL (0–150 μM) against a fixed concentration of 50 μM E7 dimer to verify this stoichiometry. The E6/E7 complex formed at a molar ratio of 1:1 in Figure 3*C* showed a sedimentation coefficient in the range of s = 6.0 to 7.0, and 89% of fl-16E7 formed a complex with ∼100 μM MBP-16E6_4C4S-LxxLL further confirmed the results obtained previously. Combining the results obtained from sedimentation velocity and sedimentation equilibrium, the broader distribution of the complex as compared with the single species might be due to the equilibrium between the 1:2 and 2:2 complex. The summary of the stoichiometry of MBP-16E6_4C4S-LxxLL, fl-16E7, and MBP-16E6_4C4S-LxxLL/fl-16E7 is shown in Table 1.

**Table 1:** MW and sedimentation coefficient of MBP-16E6_4C4S-LxxLL, fl-16E7, and MBP-16E6_4C4S-LxxLL/fl-16E7

Proteins	MW theoretical (kDa)	MW_app_ (kDa)	Sedimentation coefficient (s_app_)	Oligomeric state
MBP-16E6_4C4S-LxxLL	62	63.4 ± 4.9	4.30	Monomer (E6_1_)
fl-16E7	11	19.7 ± 2.1	1.70	Dimer (E7_2_)
MBP-16E6_4C4S-LxxLL/fl-16E7	146	142.0 ± 6.5	6.70	2 × E6 + 2 × E7

MW_app_ indicates the calculated MW.

Taken together, the results indicate that the proteins of E6 and E7 from HPV16 form the complex at a molar ratio of 2:2, and the calculated MW revealed two E7 molecules and two E6 molecules in the complex.

### HPV16 and HPV31 E6 and E7 proteins share a similar binding affinity according to FP assay

We performed FP to quantify the binding affinity of 16E6 and 31E6 to the 16E7 and 31E7, respectively, using recombinantly produced proteins (Fig. 1*C*) and fluorescein-labeled E7 as a probe.

**Figure 4:**
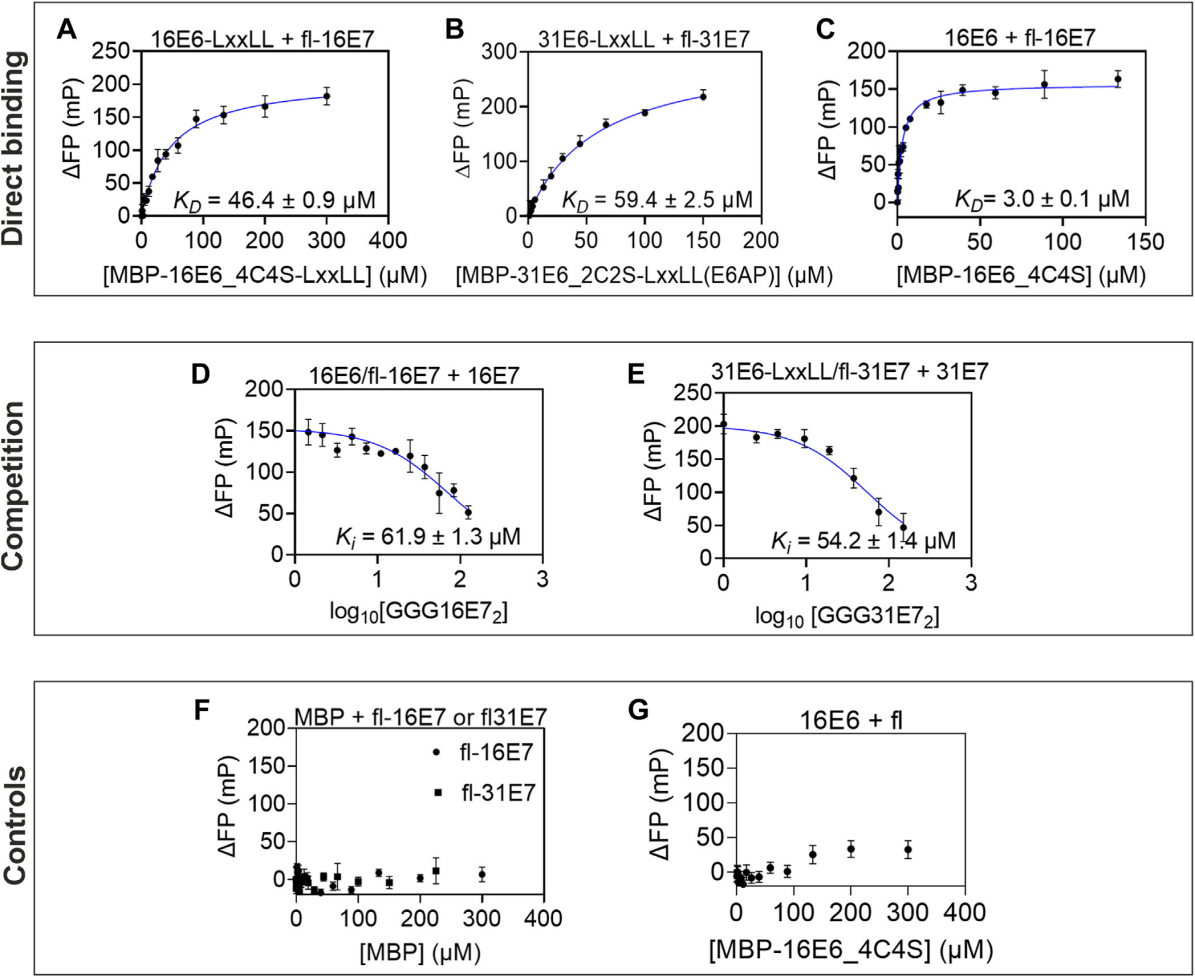
**The binding affinity of the E6–E7 complex.***A*–*C*, direct binding curves of purified MBP-E6-LxxLL or MBP-E6 with fl-E7 were monitored in fluorescence polarization by titrating fl-E7 with an increasing amount of E6. All E6 proteins used above consist of C/S mutation. The clear increase in FP indicates a binding event has occurred. The binding affinity of MBP-E6-LxxLL and fl-E7 from HPV16 (*A*) and HPV31 (*B*) is similar. MBP-16E6_4C4S (*C*) shows a 15-fold higher binding affinity than MBP-16E6_4C4S-LxxLL. *D* and *E*, the reversibility of the complex formation was monitored with a competitive measurement by titrating the complex with an increasing amount of nonlabeled GGG-E7 dimer. A decrease in the FP signal indicates the reversible complex formation. Concluding from the competition measurement, HPV16 (*D*) and HPV31 (*E*) formed E6/E7 complex at a similar binding affinity, and the binding is independent of the LxxLL peptide from E6AP. *F*, an increasing amount of MBP was titrated against fl-16E7 or fl-31E7. No significant increase in the FP signal indicates that the binding between E6 and E7 is not an artifact of the MBP tag. *G*, an increasing amount of MBP-16E6_4C4S was titrated against the fluorescein peptide. A slight increase in FP signal at higher concentrations indicates the presence of artifact from fluorescein. All FP signals were subtracted with the FP signal of respective fl-E7 or fl alone and plotted against concentrations of MBP-16E6_4C4S-LxxLL, MBP-31E6_2C2S-LxxLL, MBP-16E6_4C4S, MBP, or nonlabeled GGG-E7 as indicated. The error bar plotted is the standard deviation of the mean from three technical replicates. FP, fluorescence polarization; MBP, maltose-binding protein.

For direct binding, the MBP-E6-LxxLL was titrated 1.5-fold against a fixed concentration of fl-E7, which showed an increase in the FP signal, indicating an interaction. The binding curve fitted with one-site–specific binding fit revealed a similar affinity of 46.4 ± 0.9 μM for MBP-16E6_4C4S-LxxLL (Fig. 4*A*) and 59.4 ± 2.5 μM for MBP-31E6_2C2S-LxxLL (Fig. 4*B*), respectively. The LxxLL direct fusion does stabilize E6, especially for a p53-ready conformation (22, 23). However, it does not resemble the actual situation. Hence, we repeated the same experiment using MBP-16E6_4C4S without the LxxLL peptide of E6AP. Surprisingly, this experiment showed an affinity of 3.0 ± 0.1 μM (Fig. 4*C*).

In addition, we performed a competition assay to analyze the reversibility of the observed complex formation. For this, a two-fold dilution of unlabeled GGG-E7 dimer was prepared to compete with the E6/E7 complex formed at 60 to 80% saturation concentration. Considering the effect of the direct fusion of E6AP-LxxLL-peptide on the E6 and E7 interaction observed in direct binding, we used unlabeled GGG-16E7 dimer (titrated from 200 μM) to compete with MBP-16E6_4C4S/fl-16E7 and unlabeled GGG-31E7 dimer (titrated from 150 μM) to compete with MBP-31E6_2C2S-LxxLL/fl-31E7. We observed a decreasing FP signal in both cases, indicating a reversible complex formation of E6 and E7 (Fig. 4, *D* and *E*). In the case of MBP-31E6_2C2S-LxxLL, the direct binding and the competition showed a similar affinity of 59.4 ± 2.5 μM and 54.2 ± 1.4 μM, respectively. However, for MBP-16E6_4C4S without LxxLL fusion, the affinity obtained from direct and competitive measurement differs significantly by approximately 21-fold.

No significant binding was observed when we conducted the same experiment with the controls by substituting MBP-E6 or MBP-E6-LxxLL with MBP only (Fig. 4*F*). Because of the different affinity obtained in Figure 4, *A* and *C*, we titrated MBP-16E6_4C4S against 42 nM unconjugated fluorescein peptide (equivalent to the fluorescein concentration in fl-16E7) to investigate the effect of fluorescein in the binding. We observed a slight increase in FP signal at concentrations higher than 100 μM of MBP-16E6_4C4S (Fig. 4*G*). This result indicates that the fluorescein may impact E6 and E7 binding without the LxxLL peptide.

Taken together (Table 2), an interaction between E6 with E7 of HPV16 and HPV31 was verified. Both HPV16 and HPV31 share a similar binding affinity between E6-LxxLL and E7. Interestingly, 16E6 without a direct LxxLL fusion seems to bind stronger to E7 in direct binding but not competition.

**Table 2:** Binding affinity of MBP-E6 or MBP-E6-LxxLL with fl-E7

Complex	Direct measurement	Competition
fl-16E7/MBP-16E6_4C4S	3.0 ± 0.1 μM	61.9 ± 1.3 μM
fl-16E7/MBP-16E6_4C4S-LxxLL	46.4 ± 0.9 μM	ND
fl-16E7/MBP-31E6_2C2S-LxxLL	59.4 ± 2.5 μM	54.2 ± 1.4 μM

ND, no data.

### The CR1/2 region of E7 participates in the complex formation according to the FP assay

**Figure 5:**
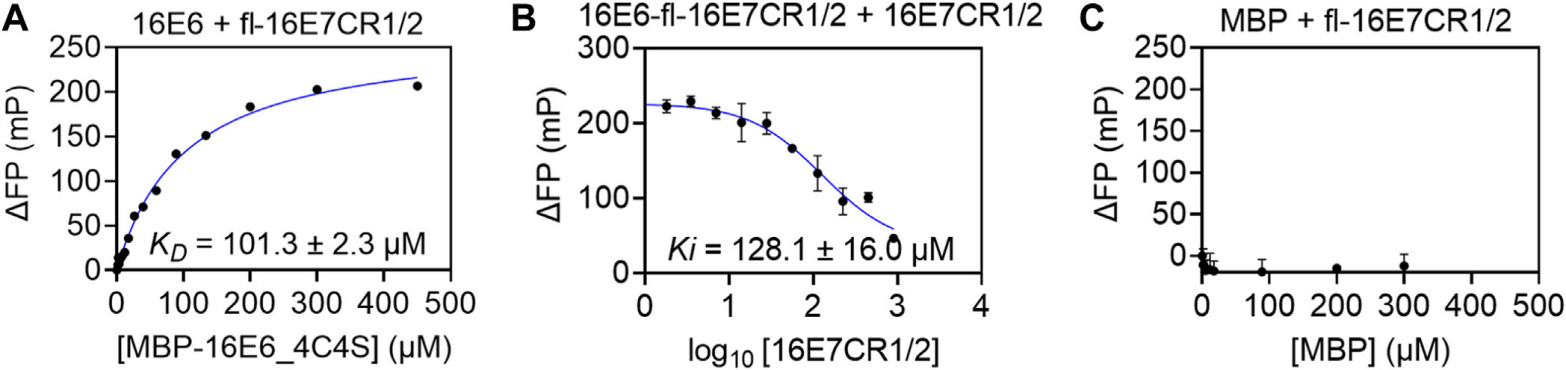
**E6 binds N terminus of E7 protein.***A*, the direct binding curve of purified MBP-16E6_4C4S with fl-16E7CR1/2 (amino acid [aa] 1–44) was monitored in fluorescence polarization (FP) by titrating fl-16E7CR1/2 (aa 1–44) with an increasing MBP-16E6_4C4S. *B*, the reversibility of the complex formation was observed with a competitive measurement by titrating the complex with an increasing amount of nonlabeled 16E7CR1/2 (aa 1–44). A decrease in the FP signal indicates the reversible complex formation. Both direct and competitive binding show similar binding affinity. *C*, an increasing amount of MBP was titrated against fl-16E7CR1/2 (aa 1–44). No significant increase in FP signal indicates that the binding between E6 and E7CR1/2 (aa 1–44) is not an artifact of the MBP tag. MBP, maltose-binding protein.

We synthesized 16E7CR1/2 (amino acids [aa] 1–44) peptide, labeled and unlabeled with fluorescein dye at N terminus. An increasing amount of MBP-16E6_4C4S was titrated against 350 nM fl-16E7CR1/2 (aa 1–44). An increase in FP signal was observed with MBP-16E6_4C4S again, indicating an interaction (Fig. 5*A*). Then, the nonlabeled 16E7CR1/2 (aa 1–44) was titrated against the complex formed using 200 μM MBP-16E6_4C4S and 350 nM fl-16E7CR1/2 (aa 1–44) (60% saturation) for competition. The decrease of the FP signal indicates a reversible complex formation (Fig. 5*B*) again. The affinity for direct binding and competition were similar, 101.3 ± 2.3 μM and 128.1 ± 16.0 μM (Fig. 5, *A* and *B*), respectively. No increase was observed in the FP signal when the fl-16E7 was titrated with an increasing amount of MBP up to 300 μM (Fig. 5*C*). This means that as full-length E7, the CR1/2 (aa 1–44) of E7 does not bind to MBP but E6. However, the affinity of the complex MBP-16E6_4C4S/16E7CR1-2 (aa 1–44) is approximately two-fold lower than MBP-16E6_4C4S/GGG16E7 but in the same micromolar range.

Hence, these results showed that E6 is binding to the N-terminal region of 16E7, the CR1/2 (aa 1–44).

## Discussion

The direct interaction between the proteins E6 and E7 of HPVs has not been described yet. Both proteins act together to immortalize keratinocytes and are overexpressed in carcinoma cells as described earlier.

Our FACS–FRET and co-IP results showed that the E6 and E7 of HPV16 and HPV31 who are the two very closely related HPV types that belong to the alpha genus, species-9 interact with each other. Furthermore, HPV18 belongs to the alpha genus, species-7; and HPV38 belongs to the beta genus, species-2 also shows the interaction between E6 and E7 in FACS–FRET. These results indicate that the interaction between E6 and E7 might be a general phenomenon across HPV phylogenetic trees. The *in vitro* study *via* analytical ultracentrifugation and FP verified the direct interaction between E6 and E7 of HPV16 and HPV31 and revealed a binding affinity of ∼55 to 60 μM. In addition, we revealed the engagement of 16E7CR1/2 (1, 2, 3, 4, 5, 6, 7, 8, 9, 10, 11, 12, 13, 14, 15, 16, 17, 18, 19, 20, 21, 22, 23, 24, 25, 26, 27, 28, 29, 30, 31, 32, 33, 34, 35, 36, 37, 38, 39, 40, 41, 42, 43, 44) in the complex formation.

It is known that E7 is a highly stable dimer and the most prominent oligomeric species under physiological conditions (9, 13, 34). Accordingly, our AUC data revealed that the fl-16E7 protein (Fig. 3) is a homogenous dimer under tested conditions, whereas MBP-16E6_4C4S-LxxLL is a monomer. In addition, the AUC complex analysis of fl-16E7 and MBP-16E6_4C4S-LxxLL revealed that two molecules of MBP-16E6_4C4S-LxxLL and two molecules of fl-16E7 are forming the predominant species at a 2:2 ratio of E6:E7. Being E7 a highly stable dimer, we propose that an fl-16E7 dimer binds two molecules of MBP-16E6_4C4S-LxxLL. The observed minor intermediate species have also appeared, which could indicate a ratio of 1:2 (1× MBP-16E6_4C4S-LxxLL and 2× fl-16E7 molecules).

Because of the observed intermediate species in AUC, we fitted the FP data with a cooperative binding model, but the results were inconclusive (data not shown). Therefore, all the binding curves and affinities shown were based on the one-site–specific binding model, which resembles the average over the two binding sites. The similar affinities observed in HPV16 and HPV31 (Fig. 4, *D* and *E*) indeed draw interest in comparing the affinities of E6/E7 from other HPV genera. However, because of inefficient material availabilities of recombinant E6 and E7, the *in vitro* analysis for the stoichiometry and affinities measurement was limited to HPV16 and HPV31.

Notably, the affinity obtained in Figure 4, *A* and *C* differs by ∼15-fold, and the fluorescein seems to exert an artifact binding with MBP-16E6_4C4S. The major difference between the two is the fusion of the LxxLL motif directly to the C terminus of 16E6, which binds to the hydrophobic LxxLL binding pocket and stabilizes the E6 (22, 23, 35). We hypothesize that the exposure of the LxxLL hydrophobic binding site in MBP-16E6_4C4S might bind to the fluorescein, thus contributing to the higher affinity in Figure 4*C*. These data also show the importance of employing a competitive measurement in verifying and concluding the binding affinities of the two proteins. The competitive measurement showed a similar affinity for GGG-16E7/MBP16E6_4C4S compared with GGG-31E7/MBP-31E6_2C2S-LxxLL (Fig. 4, *D* and *E*). These results suggest that the LxxLL peptide of E6AP may not interact with E7 but rather impair the binding to the fluorescein. Moreover, it was shown that the E6N and E6C domains are rather flexible (35) and are held in place by the E6AP LxxLL peptide to facilitate the p53 binding (22). Hence, we hypothesized that in the absence of the LxxLL fusion, the conformational flexibility of the E6N and E6C may also impact its binding to the E7.

Detailed 3D-structural information of full-length E7 is unavailable on Protein Data Bank, presumably because of its structural flexibility caused by the highly disordered N-terminal region (10). The binding affinity of the 16E7CR1/2 (aa 1–44) to MBP-16E6_4C4S is in the same range but is two-fold lower than the full-length GGG-16E7. We conducted an additional competition to compete for the fl-16E7/MBP-16E6_4C4S complex with nonlabeled 16E7CR1/2. We revealed an affinity of 288.0 ± 7.8 μM, almost a five-fold difference showing that the 16E7CR1/2 (1, 2, 3, 4, 5, 6, 7, 8, 9, 10, 11, 12, 13, 14, 15, 16, 17, 18, 19, 20, 21, 22, 23, 24, 25, 26, 27, 28, 29, 30, 31, 32, 33, 34, 35, 36, 37, 38, 39, 40, 41, 42, 43, 44) has a lower binding affinity (Fig. S5) than full-length 16E7. These observations might be due to its intrinsically disordered properties. It has been described that a protein’s intrinsically disordered proteins or regions exert low affinity to their ligands (36, 37). The idea of low affinity was derived from the coupled folding-binding process whereby the net free-energy change during the folding (free energy increases), and their binding to the ligand (free energy decreases) is smaller than in a pure binding process (37, 38). Another hypothesis for this observation is that 16E7CR1/2 (1, 2, 3, 4, 5, 6, 7, 8, 9, 10, 11, 12, 13, 14, 15, 16, 17, 18, 19, 20, 21, 22, 23, 24, 25, 26, 27, 28, 29, 30, 31, 32, 33, 34, 35, 36, 37, 38, 39, 40, 41, 42, 43, 44) may not be the only binding region. Nevertheless, 16E7CR1/2 (1, 2, 3, 4, 5, 6, 7, 8, 9, 10, 11, 12, 13, 14, 15, 16, 17, 18, 19, 20, 21, 22, 23, 24, 25, 26, 27, 28, 29, 30, 31, 32, 33, 34, 35, 36, 37, 38, 39, 40, 41, 42, 43, 44) is definitely one binding region for MBP-16E6_4C4S. Regarding the details of complex formation, a structural analysis would be necessary to understand the association mechanism between E6 and E7.

The synergistic effects of E6 and E7 in developing and maintaining HPV-associated carcinogenesis have been well reviewed (5, 6). HPV E7 protein does it by inhibiting pRb (11, 12), whereas HPV E6 protein does it by degrading p53 (24), in which these two models are the most studied. Moreover, E6 and E7 also interfere with cellular pathways essential for immune invasion (39). It is proposed that the inhibition effect on NF-κB activity is essential for the initial HPV infection. As soon as the transformation of epithelial cells occurs, the NF-κB is activated and might promote tumorigenesis (6). It was previously shown that the expression of E7 and both E6 and E7 downregulate the basal and tumor necrosis factor-α–induced NF-κB activity in the cervical transformation zone where most cervical cancers start to develop; the effect of E6 on tumor necrosis factor-α–induced NF-κB activity is rather mild (40, 41). On the other hand, several studies observed the upregulation of NF-κB activity in developed cervical carcinomas (42, 43, 44) where E6 and E7 are highly expressed. Besides, E6 and E7 inhibit Scrib and PTPN14, respectively to activate the Yes-association protein (YAP1), thus inducing the Hippo signaling pathway that drives cellular proliferation (45, 46). Furthermore, angiogenesis is driven by E6 and E7 by activating proangiogenic factors, oxygen-sensitive transcriptional activator hypoxia-inducible factor-1 (47) and vascular endothelial growth factor (48, 49, 50), though the mechanism is not well understood.

**Figure 6:**
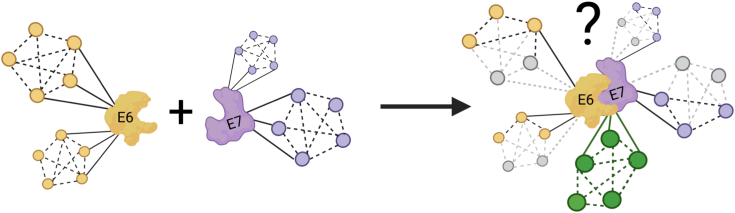
**Schematic diagram illustrating putative roles of the complex of E6 with E7.** Whether E6 or E7 may retain (*black solid and dotted lines* connecting *yellow* or *violet* interactomes), loss (*gray dotted lines* connecting *gray* interactomes), or gain (*green* interactome network) the ability to target cellular factors upon complex formation is discussed. Figure created with Biorender.com.

With the interaction of E6 with E7 observed here, it is conceivable that the complex may be formed to maintain a network balance between free *versus* complexed E6 and E7 to allow the targeting of different cellular proteins and pathways at distinct time points during the infectious cycle. It could be that the interaction between E6 and E7 may be necessary to support viral replication, immune evasion, and tumorigenesis rather than a synergistic interplay between the single activities of E6 and E7 as has been assumed so far. Further studies are required to unravel whether the complex may facilitate, enhance, or dismiss the binding to the already known cellular targets or even allow the gaining of new targets (Fig. 6). Our findings provide a new perspective for studying the molecular mechanism of E6 and E7 in the viral life cycle, cellular transformation, and carcinogenesis.

## Experimental procedures

### Constructs

For co-IP, HPV16 E6, HPV31 E6, HPV16 E7, and HPV31E7 (PAVE reference number HPV16REF.1/GI:333031; HPV31REF.1/GI:333048) plasmid constructs were obtained from GenScript, whereby they were cloned in pcDNA3.1 vector with E6s as untagged constructs, whereas E7s were fused to the triple human influenza HA epitopes (3xHA) at their amino (N) terminus with an SG linker. For FACS–FRET, HPV 16E6, 31E6, 18E6, 6E6, and 38E6 constructs were cloned in pmTagBFP-C1. In contrast, HPV 16E7, 31E7, 18E7, 6E7, and 38E7 were cloned into pEYFP-C1 (PAVE reference number HPV18REF.1/GI:60975, HPV6REF.1/GI:60955, HPV38REF.1/GI:1020234) *via* restriction cloning or Gibson cloning obtaining an N-terminal fusion of E6 with mTagBFP2 or N-terminal fusion of E7 with EYFP with an SG linker. For recombinant protein production in *Escherichia coli*, it is known that E6 exerts solubility issues, as reviewed (51). Hence, to conquer this obstacle, we fused MBP to the N terminus of E6 protein to increase the solubility and the LxxLL peptide sequence of E6AP (ESSELTLQELLGEER) to the C terminus as it is known to bind and stabilize E6 proteins. Two linkers were cloned upstream and downstream of the E6 sequences, respectively. In addition, the mutations of the nonconserved surface-exposed cysteine to serine were introduced in the E6 proteins to overcome oxidation and disulfide-mediated oligomerization, four cysteines were mutated for 16E6 (C80, C97, C111, and C140) and two for 31E6 (C97 and C111). HPV16 E7 and HPV31 E7 obtained from GenScript were cloned in the pET28a vector to obtain the N-terminally fused hexa-histidine (His_6_) constructs with additional tobacco etch virus (TEV) cleavage site and a GGG linker cloned upstream of the E7 that allows tag cleavage by TEV protease followed by *sortase A*-based protein labeling. The detailed expression and purification methods for E6 and E7 full-length proteins can be found in Supplementary information SI7. HPV16E7CR1/2 (aa 1–44) was synthesized as described in Supplementary information SI7. An overview of all constructs used is shown in Figure 1. All E6 and E7 constructs mentioned are aligned with the protein sequences obtained from PAVE database unless mutations are stated (PAVE reference number HPV16REF.1/GI:333031; HPV31REF.1/GI:333048; HPV18REF.1/GI:60975, HPV6REF.1/GI:60955, and HPV38REF.1/GI:1020234). The UniProtKB accession number and protein sequences of the E6 and E7 proteins from each HPV type are also listed in Table S1 in Supporting Information SI1.

### Cell culture

HPV-negative cervical cancer cell line C33A was cultured in Dulbecco’s modified Eagles’ medium (Gibco; catalog no.: 41965-062) supplemented with 10% fetal bovine serum (Gibco; catalog no.: 10270-106) and gentamicin (50 μg/ml) (Gibco; catalog no.: 157710049) at 37 °C, 95% humidity, and 5% carbon dioxide. One day before transfection, 200,000 cells/well were seeded in a 12-well plate (Thermo Scientific; catalog no.: 150628) or 6,000,000 cells in a 150 mm sterile cell culture plate (Thermo Scientific; catalog no.: 168381). The cells were transfected with respective plasmid DNA using jetPRIME (Polyplus; catalog no.: 101000046) following the manufacturer’s instructions on day 2. Cells were trypsinized with Gibco trypsin–EDTA (Gibco; catalog no.: 25200072) for FACS measurements or lyse for co-IP 48 h post-transfection.

### FACS–FRET

C33A cells coexpressing mTagBFP2-E6 and EYFP-E7 (Fig. 1) were used in FACS–FRET measurements. The positive control mTagBFP2-EYFP direct fusion and the negative controls including the pairs of (i) mTagBFP2 + EYFP, (ii) mTagBFP2-E6s + EYFP, or (iii) EYFP-E7 + mTagBFP2 were constantly employed in the measurement and analysis to ensure appropriate gating as described previously (52). All cells were washed in precooled FACS buffer (Dulbecco′s PBS with 1% v/v fetal bovine serum) and resuspended in 250 μl of FACS buffer, followed by FACS measurement using MACSQuant VYB Flow Cytometer (Miltenyi Biotec). FACS–FRET measurement was performed as described earlier (52) but briefly: Cells expressing fluorescent proteins mTagBFP2 and EYFP were detected in channel V1 (405/450 (50)) nm and B1 (488/529 (50)) nm, respectively. FRET signal was assessed in channel V2 (405/525 (50)) nm. FACS and statistical analysis were conducted using FlowLogic, version 7.2.2 (Miltenyi–Inivai) and GraphPad Prism (GraphPad Software, Inc), version 9.1.2 (226), respectively. All figures presented were prepared using CorelDrawX7, version 17.5.0.907 from Alludo (formerly Corel Corporation).

### Co-IP

The HA IP was performed with extracts from C33A cells that coexpressed the 3xHA-E7 and the untagged E6 proteins 48 h post-transfection. The cells were treated with 1 μM MG-132 proteasome inhibitors (AdipoGen Life Sciences; AG-CP3-0011) 16 h before harvesting. Cells from four 150 mm plates with 90% confluency were harvested in 3 ml of lysis buffer (10% [v/v] glycerol [MP Biomedicals; catalog no.: 4800689]; 50 mM Hepes, pH 7.5 [Carl Roth; catalog no.: 9105.4]; 3 mM magnesium chloride [Merck; catalog no.: 105833]; 0.1% [v/v] IGEPAL CA-630 [NP-40] [Merck; catalog no.: 18896]; 150 mM sodium chloride [NaCl] [Carl Roth; catalog no.: 3957.2]; 1 mM Tris(2-carboxyethyl)phosphine [TCEP] [Alfa Aesar; catalog no.: J60316]; 200 μM zinc chloride [Carl Roth; catalog no.: 3533]; supplemented with 1 μl benzonase endonuclease [Merck; catalog no.: 101656] per 10 ml buffer, one tablet PhosSTOP [Roche; PHOSSRO] per 25 ml buffer, and one tablet of cOmplete EDTA-free Protease Inhibitor Cocktail [Roche; catalog no.: COEDTAF-RO] per 50 ml buffer) prior use. Cell lysates were incubated on a shaker in the cold room (4–8 °C) for 1 h before centrifuging at 18,000*g* at 4 °C for 10 min to remove cell debris and unlysed cells. Bradford assay was conducted for the supernatant to determine the total protein concentration of the cleared crude lysates. Each time, 6000 μg of total protein of the cleared crude lysates were incubated with 50 μl of anti-HA Microbeads (μMACS HA Isolation Kits [Miltenyi Biotec; catalog no.: 130-091-122]) for 2 h in the cold room (4–8 °C). Then, the suspension was loaded on μColumns (Miltenyi Biotec; catalog no.: 130-042-701), attached to a μMACS Separator (Miltenyi Biotec; catalog no.: 130-042-602), followed by five times washing steps, each time with 500 μl lysis buffer. Then, native elution with 3xHA peptide was performed to eliminate nonspecific-bound proteins as previously described (33). Proteins of interest obtained from native elution were diluted in reducing SDS-sample buffer and heated at 95 °C for 10 min before further analysis by immunoblotting.

### Immunoblotting

The proteins of interest were resolved on reducing 8 to 20% gradient SDS-PAGE gel. Proteins were electrotransferred onto nitrocellulose membrane (GE Healthcare) *via* wet blotting using blotting buffer (2.2 g/l 3-(cyclohexylamino)-1-propanesulfonic acid [Sigma; catalog no.: C2632], 0.001% [w/v] SDS [Carl Roth; catalog no.: 2326.2], 10% [v/v] methanol [Honeywell; catalog no.: 32213], pH 10.3 at room temperature) for 1 h at 70 V (constant). The membrane was blocked with 5% (w/v) albumin bovine fraction V (Serva; catalog no.: 11930) in PBS for 1 h at room temperature and probed with appropriate primary antibodies, which were all diluted in PBS with 0.1% v/v Tween-20 (Sigma; catalog no.: P9416) overnight at cold room (4–8 °C). The used primary antibodies include anti-HA Rabbit (Cell Signaling; catalog no.: 3724) for detection of 3xHA-E7 at dilution of 1:1000 and anti-16E6 (AVC #G6, Lot #15) as well as anti-31E6 (AVC #C8, Lot #8) (generously provided by Arbor Vita Corporation) at dilution of 1:10,000 for detection of 16E6 and 31E6, respectively. GAPDH was used as a loading control and detected by anti-GAPDH (6C5) from Santa Cruz Biotechnology (catalog no.: sc32233) at a dilution of 1:500. All membranes were washed three times with PBS with 0.05% v/v Tween-20 after overnight incubation with primary antibodies. Secondary antibodies IRDye 680RD Goat anti-Rabbit IgG (H + L) or IRDye 680RD Goat antimouse IgG (H + L) (LI-COR Biotechnology GmbH) were used at a dilution of 1:10,000 and incubated for 30 min at room temperature. The membrane was washed three times with PBS with 0.05% v/v Tween-20. The signal of the respective protein was then visualized using LI-COR Odyssey Fc (700 nm channel) and analyzed with Image Studio Lite Software from LI-COR Biosciences.

### Analytical ultracentrifugation

To analyze the molecular mass and the stoichiometry of the complex, we performed analytical ultracentrifugation in an analytical ultracentrifuge XL-I (Beckman Coulter) and an An-50 Ti rotor with double sector cells using the complex of fl-16E7 and MBP-16E6_4C4S-LxxLL (E6AP) formed in the assay buffer (20 mM Hepes [pH 7.5], 200 mM NaCl, and 1 mM TCEP). The absorbance at 495 nm, specific for fluorescein and therefore measures fl-16E7, was monitored. To determine the sedimentation velocity, the fl-16E7 and MBP-16E_64C4S-LxxLL proteins were mixed in a 1:1 molar ratio, and the sedimentation at 40,000 rpm, 20 °C, was analyzed for 5 h. Scans were taken every 10 min. The molecular mass was measured at 12,000 or 18,000 rpm in sedimentation equilibrium runs at 100 μM MBP-16E6_4C4S-LxxLL (measured at 280 and 301 nm), 100 μM of fl-16E7, and 100 μM complex (mixture of E6 and E7 at 1:1 molar ratio) in the assay buffer at 20 °C. Every sedimentation equilibrium was measured for at least 40 h until equilibrium was reached, with scans taken every 5 h. Equilibrium was proven experimentally with the final three scans being identical. The MBP-16E6_4C4S-LxxLL proteins were also titrated (0–150 μM) against 100 μM fl-16E7 proteins. The program SEDFIT version 12.52 from National Institutes of Health was used for data analysis (53).

### FP

For FP direct measurements, a 1.5-fold dilution series of the MBP-E6 proteins were prepared in the FP assay buffer (20 mM Hepes [pH 7.5], 200 mM NaCl, 1 mM TCEP, and 0.005% Tween-20). For each dataset shown, three technical replicates of an identical dilution series were prepared and mixed with 350 nM or 200 nM fl-E7 of HPV16 or HPV31, respectively. Finally, 50 μl of E6/E7 complexes were transferred to 96-well microplates (nonbinding microplate, 96 wells; Greiner Bio-One; catalog no.: 655900) for measurement at the multimode reader Tristar^2^ LB 942 (Berthold Technologies) equipped with a polarizer filter, with each measurement consists of 16 different protein concentrations (whereas one contained no E6 protein and corresponded to the free fl-E7). In competitive FP measurements, the E6 protein and fl-E7 were mixed in the FP assay buffer to achieve a complex formation of 60 to 80% at concentrations based on the titration of direct binding, 70 μM of MBP-31E6_2C2S-LxxLL for HPV31 and 50 μM of MBP-16E6_4C4S for HPV16, respectively. Then, a dilution series of the nonfluorescent competitor, the unlabeled HPV16 GGG-E7 and HPV31 GGG-E7 dimer proteins, were titrated against the complex. The competitive measurement was carried out identically to the direct experiment described previously. Analyses of all FP experiments were carried out in GraphPad Prism, version 9.1.2 (226). All data were fitted using the one-site–specific binding model.

## Data availability

All data are contained within the article.

## Supporting information

This article contains supporting information (54, 55, 56, 57, 58).

## Conflict of interest

The authors declare that they have no conflicts of interest with the contents of this article.
