# Supplementary material for: Evidence for direct interaction between the oncogenic proteins E6 and E7 of high-risk human papillomavirus (HPV)
Source: J Biol Chem. 2023 Jun 23;299(8):104954. doi: 10.1016/j.jbc.2023.104954 (PMC10372912; doi:10.1016/j.jbc.2023.104954)
Supplement: Supporting information [file mmc1.pdf]

Evidence for direct interaction between the oncogenic proteins E6 and E7 of high-risk Human Papillomavirus (HPV)

JiaWen Lim[1], Hauke Lilie[3], Hubert Kalbacher[4], Nora Roos[1], Desiree Isabella Frecot[1], Maximilian Feige[1], Marcel Conrady[1], Tobias Votteler[1], Alexandra Cousido-Siah[2], Giada Corradini Bartoli[1], Thomas Iftner[1], Gilles Trave[2], Claudia Simon[1*]

Affiliations:

1 Institute of Medical Virology and Epidemiology of Viral Diseases, University Hospital Tuebingen, Germany

2 Equipe Labellisée Ligue 2015, Department of Integrative Biology, Institut de Génétique et de Biologie Moléculaire et Cellulaire, CNRS, INSERM, UdS, Illkirch, France

3 Institute of Biochemistry and Biotechnology, Martin-Luther-University Halle-Wittemberg, Germany

4 Interfaculty Institute of Biochemistry, Eberhard-Karls-University Tuebingen, Germany

*Corresponding author:

Claudia.simon@med.uni-tuebingen.de

Thomas.Iftner@med.uni-tuebingen.de

List of supporting information

Supporting information	Title	Page number
SI1	Protein sequences of the E6 and E7	S-2
SI2	FACS-FRET analysis	S-3 – S-5
SI3	% signal and number of double positive cells for FACS-FRET	S-6 – S-8
SI4	CoIP full blot	S-9
SI5	FP – Competition of 16E7CR1/2 with fl-16E7/MBP-16E6_4C4S	S-10
SI6	Sedimentation profile from AUC-SV measurement	S-11
SI7	Methods of proteins expression, proteins purification, proteins labeling and peptides synthesis	S12 – S16
References		S-17

Supporting information 1 (SI1): Protein sequences of the E6 and E7

Table S1: The UniProtKB accession number and protein sequences of all mentioned E6 and E7 proteins

Proteins	UniProtKB accession number	Protein sequences
HPV16E6	P03126	MFQDPQERPRKLPQLCTELQTTIHDIILECVYCKQQLLRRE VYDFAFRDLCIVYRDGNPYAVCDKCLKFYSKISEYRHYCYS LYGTTLEQQYNKPLCDLLIRCINCQKPLCPEEKQRHLDKKQ RFHNIRGRWTGRCMSCCRSSRTRRETQL
HPV16E7	P03129	MHGDTPTLHEYMLDLQPETTDLYCYEQLNDSSEEEDEIDG PAGQAEPDRAHYNIVTFCCKCDSTLRLCVQSTHVDIRTLE DLLMGTLGIVCPICSQKP
HPV18E6	P06463	MARFEDPTRRPYKLPDLCTELNTSLQDIEITCVYCKTVLELT EVFEFAFKDLFVVYRDSIPHAACHKCIDFYSRIRELRHYSD SVYGDTLEKLTNTGLYNLLIRCLRCQKPLNPAEKLRHLNEK RRFHNIAGHYRGQCHSCCNRARQERLQRRRETQV
HPV18E7	P06788	MHGPKATLQDIVLHLEPQNEIPVDLLCHEQLSDSEEENDEI DGVNHQHLPARRAEPQRHTMLCMCCKCEARIELVVESSA DDLRAFQQLFLNTLSFVCPWCASQQ
HPV31E6	P17386	MFKNPAERPRKLHELSSALEIPYDELRLNCVYCKGQLTETE VLDFAFTDLTIVYRDDTPHGVCTKCLRFYSKVSEFRWYRY SVYGTTLEKLTNKGICDLLIRCITCQRPLCPEEKQRHLDKKK RFHNIGGRWTGRCIACWRRPRTETQV
HPV31E7	P17387	MRGETPTLQDYVLDLQPEATDLHCYEQLPDSSDEEDVIDS PAGQAEPDTSNYNIVTFCCQCKSTLRLCVQSTQVDIRILQE LLMGSFGIVCPNCSTRL
HPV6E6	P06462	MESANASTSATTIDQLCKTFNLSMHTLQINCVFCKNALTTA EIYSYAYKHLKVLFRGGYPYAACACCLEFHGKINQYRHFD YAGYATTVEEETKQDILDVLIRCYLCHKPLCEVEKVKHILTK ARFIKLNCTWKGRCLHCWTTCMEDMLP
HPV6E7	P06464	MHGRHVTLKDIVLDLQPPDPVGLHCYEQLVDSSEDEVDEV DGQDSQPLKQHFQIVTCCCGCDSNVRLVVQCTETDIREV QQLLLGTLNIVCPICAPKT
HPV38E6	Q80907	MELPKPQTVQQLSDKLTVPVEDLLLPCRFCNSFLTYIELRE FDYKNLQLIWTQEDFVFACCSSCAYASAQYECQQFYELTV FGREIEQVEQQTIGLIVIRCQYCLKCLDLIEKLDICCSHQAFH KVRGNWKGRCRHCKAIE
HPV38E7	Q80908	MIGKQATLRDIVLEELVQPIDLHCHEELPDLPEDIEASVVEE EPAYTPYKIIVLCGGCEVRLKLYVWATDAGIRNLQDCLLGD VRLLCPTCREDIRNGGR

Supporting information 2 (SI2): FACS-FRET analysis

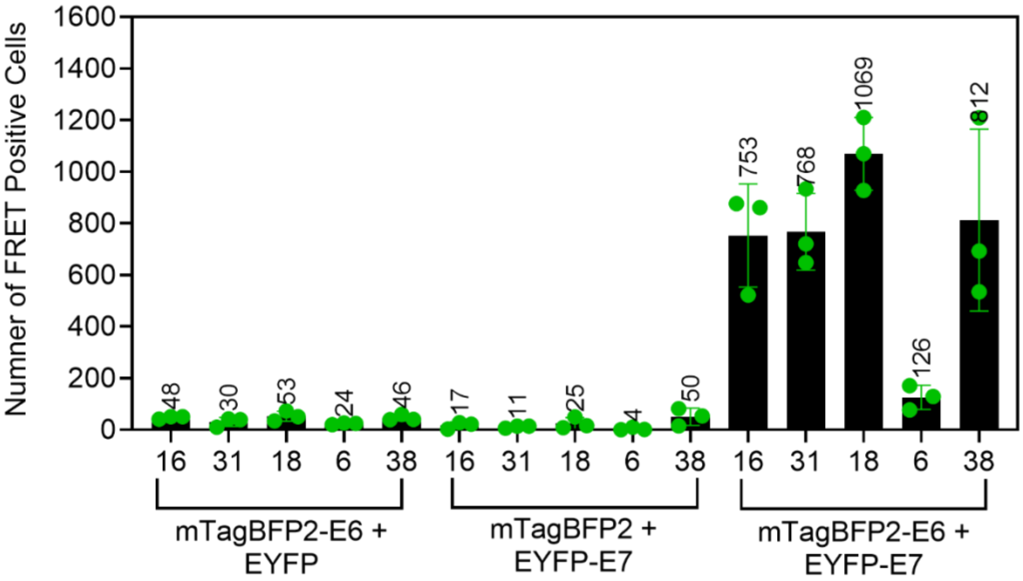

Figure S2. E6 interacts with E7 FACS-FRET assay. C33A co-expressing mTagBFP2-E6 and EYFP-E7 were subjected to FACS-FRET. The low number of FRET-positive cells in the controls of mTagBFP-E6 + EYFP and mTagBFP2 + EYFP-E7 indicate no interaction seen. The E6 and E7 proteins from high-risk alpha HPV16, HPV31, HPV18, and beta HPV38 showed positive cells above the threshold of 500 cells. The number of FRET-positive cells for HPV6 is below the threshold; thus, the interaction is unclear. Data are derived from the mean value of three independent biological replicates. The error bars are plotted to represent the standard deviation of the mean value from the three independent biological replicates. The green dots represent the scatter dot plot of the three independent biological replicates. Please see Table S2B for detailed statistical data.

Table S2A: Statistical analysis data for Figure 2

FRET pair	HPV type	*n*	Mean	Standard deviation	p-value
mTagBFP2-E6 + EYFP	16	3	1.0	0.1	NA
	31	3	0.9	0.5	NA
	18	3	1.0	0.2	NA
	6	3	2.8	1.7	NA
	38	3	1.3	0.2	NA
mTagBFP2- + EYFP-E7	16	3	0.1	0.1	NA
	31	3	0.1	0.0	NA
	18	3	0.1	0.1	NA
	6	3	0.0	0.0	NA
	38	3	0.4	0.2	NA
mTagBFP2-E6 + EYFP-E7	16	3	13.0	1.7	0.006
	31	3	16.3	2.3	0.006
	18	3	15.0	1.2	0.002
	6	3	8.6	3.4	0.049
	38	3	17.9	2.5	0.006

*NA indicates not applicable

Table S2B: Statistical analysis data for Figure S2

FRET pair	HPV type	*n*	Mean	Standard deviation
mTagBFP2-E6 + EYFP	16	3	47	5
	31	3	30	17
	18	3	52	19
	6	3	24	3
	38	3	46	11
mTagBFP2 + EYFP-E7	16	3	17	13
	31	3	11	4
	18	3	24	22
	6	3	3	5
	38	3	50	33
mTagBFP2-E6 + EYFP-E7	16	3	753	200
	31	3	767	148
	18	3	1069	141
	6	3	125	47
	38	3	812	352

Supporting information 3 (SI3): % signal and number of double positive cells for FACS-FRET

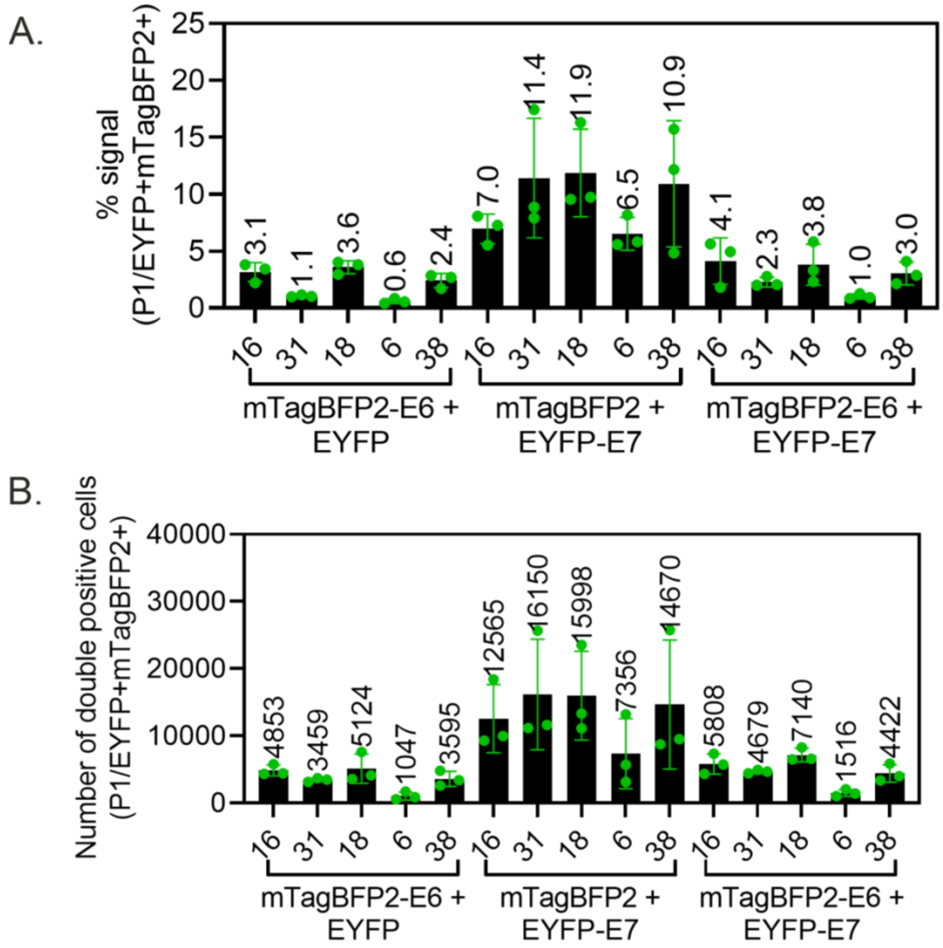

Figure S3. % signal and the number of double positive cells.
C33A co-expressing the mTagBFP2-E6 and EYFP-E7, as indicated, are subjected to FACS-FRET measurement. Viable cells are gated for EYFP and mTagBFP2 to examine cells expressing both mTagBFP-E6 and EYFP-E7 in % signal (**A.**) and the number of cells (**B.**) Data are derived from the mean value of three independent biological replicates with the mean value stated above each bar. The error bars are plotted to represent the standard deviation of the mean value from the three independent biological replicates. The green dots represent the scatter dot plot of the three independent biological replicates. Please see Table S3A and Table S3B for detailed statistical data.

Table S3A. Statistical analysis data for Figure SI3A

FRET pair	HPV type	*n*	Mean	Standard deviation
mTagBFP2-E6 + EYFP	16	3	3.1	0.8
	31	3	1.1	0.1
	18	3	3.6	0.6
	6	3	0.6	0.2
	38	3	2.4	0.6
mTagBFP2- + EYFP-E7	16	3	7.0	1.3
	31	3	11.4	5.3
	18	3	11.9	3.8
	6	3	6.5	1.4
	38	3	10.9	5.6
mTagBFP2-E6/EYFP-E7	16	3	4.1	2.0
	31	3	2.3	0.4
	18	3	3.8	1.8
	6	3	1.0	0.2
	38	3	3.0	1.0

Table S3B. Statistical analysis data for Figure SI3B

FRET pair	HPV type	*n*	Mean	Standard deviation
mTagBFP2-E6 + EYFP	16	3	4853	790
	31	3	3459	266
	18	3	5123	2184
	6	3	1047	588
	38	3	3595	1115
mTagBFP2 + EYFP-E7	16	3	12565	5076
	31	3	16150	8223
	18	3	15998	6611
	6	3	7356	5227
	38	3	14670	9597
mTagBFP2-E6 + EYFP-E7	16	3	5808	1505
	31	3	4678	261
	18	3	7140	997
	6	3	1515	504
	38	3	4422	1292

Supporting information 4 (SI4): CoIP full blot

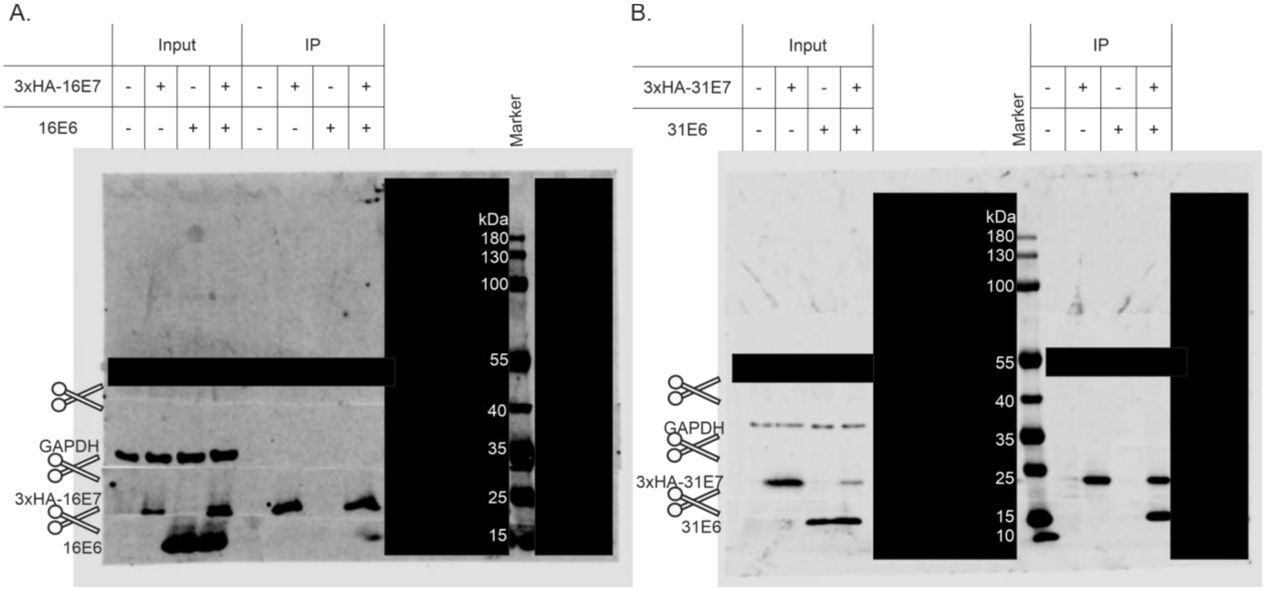

Figure S4. CoIP full blot. 70 µg cell lysates from C33A cells (input) or 25 µL of proteins precipitated with α-HA antibody (IP) were subjected to immunoblot analysis. The membrane blot was spliced after Western blotting to probe with several antibodies. First, the blots spliced above 15 kDa marker bands were probed with anti-16E6 and anti-31E6 antibodies, respectively. Then, the blots with splice site below 35 kDa marker band was probed with anti-HA antibodies for the signal of 3xHA-16E7 and 3xHA-31E7 followed by anti-GAPDH probing for the blots at splice site above 40 kDa. The antibodies probing membrane blot above the 40 kDa marker band are irrelevant for this manuscript. All spliced membranes were put together and aligned following molecular marker bands after probing with respective antibodies. Later, the aligned membrane strips were visualized at the same time at LI-COR Odyssey Fc as a single image. For presentation, the membrane blot shown in Figure 2 was cropped directly above 40 kDa marker band. The untagged E6 of alpha high-risk HPV16 **(A.)** or HPV31 **(B.)** were co-immunoprecipitated with 3xHA-16E7 or 3xHA-31E7, respectively. Irrelevant data on the same blot were covered in black.

Supporting information 5 (SI5): FP – Competition of 16E7CR1/2 with fl-16E7/MBP-16E6_4C4S

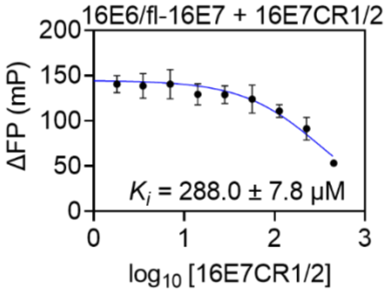

Figure S5. E6 binds the N-terminus of the E7 protein at a lower affinity. The reversibility of the fl-16E7/MBP-16E6_4C4S complex formation was observed with a competitive measurement by titrating the complex with an increasing amount of non-labeled 16E7CR1/2 (aa 1-44). A decreased FP signal indicates the reversible complex formation confirming an interaction. The binding is almost 5-fold lower compared to the GGG-16E7 FL.

Supporting information 6 (SI6): Sedimentation profile from AUC-SV measurement

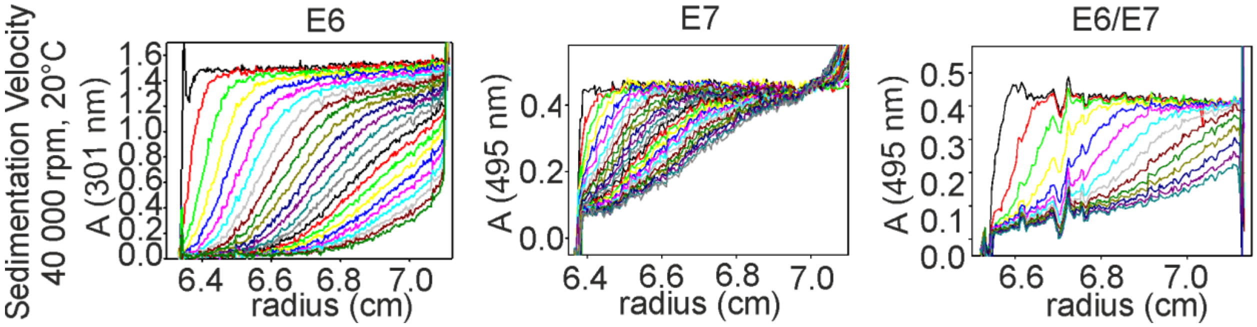

Figure S6. Sedimentation profile from sedimentation velocity measurement. MBP-16E6_4C4S-LxxLL was monitored at 301 nm wavelength. The fl-16E7 and the complex were monitored at 495 nm wavelength for the signal of the fluorescein. The shift in the sedimentation profiles indicates a binding event between MBP-16E6_4C4S-LxxLL and fl-16E7.

Supporting information 7 (SI7): Methods of proteins expression, proteins purification, proteins labeling and peptides synthesis

Protein expression

All E6 proteins were produced in *E. coli* BL21(DE3) at 16 °C after the addition of 100 µM $ZnCl_2$ and induction with 1 mM isopropyl-β-d-thiogalactopyranoside (IPTG) (MP Biomedicals, 02194029) overnight at an optical density at 600 nm (OD_{600}) of ~0.8 in Terrific Broth (TB) medium (12 g/L tryptone, 24 g/L yeast extract, 4 ml/L glycerol, 5 g/L NaCl, 0.017 M KH2PO4, and 0.072 M K2HPO4) with 30 µg/mL kanamycin as a selective antibiotic. All E7 proteins were expressed in *E. coli* BL21(DE3) using Bioreactor Labfors (INFORS AG, Bottmingen) at 20 °C in an M9 medium, following the instructions described previously in (1). MBP proteins were produced in *E. coli* BL21 and *Sortase A* (*Srt A*) in *E. coli* Rosetta 2 and expressed in the TB medium at 37°C. The expression was induced with 1 mM IPTG at an optical density at 600 nm (OD_{600}) of ~0.8 in TB using 100 µg/mL of Ampicillin as a selective antibiotic. Chloramphenicol was used additionally at 20 µg/mL for *Srt A* production in Rosetta 2. TEV protease was produced in *E. coli* BL21(DE3) and expressed in the TB medium at 37 °C. The expression was induced with 1 mM IPTG at an optical density at 600 nm (OD_{600}) of ~0.8 in TB using 30 µg/mL kanamycin as a selective antibiotic. The expression cultures were allowed to grow to OD_{600} of ~6.0 and harvested by centrifugation at 8 000 × *g* for 20 minutes at 4 °C.

Protein purification

The harvested *E. coli* biomass for production of the different E6 and E7 proteins were resuspended in 25 mL lysis buffer per 10 g wet biomass [50 mM Tris-HCl, pH 8 at 8 °C, 300 mM NaCl, 10 mM $MgCl_2$, 10 µM $ZnCl_2$, 5 % v/v glycerol, 1 mM TCEP] and approximately 4 mg of lysozyme (Carl Roth, #8259), 1 µL Benzonase® Endonuclease per 2 g wet biomass, 1 tablet cOmpleteTM EDTA-free Protease Inhibitor Cocktail per 50 mL buffer were added and incubated with gentle stirring for one hour in the cold room (4 - 8 °C). The cells were lysed by French press (Thermo Spectronic French Pressure Cell Press Model FA-078 With Pressure Cell) in 3 cycles at 600–1000 bar pressure. The lysate was centrifuged (1 hour at 30 000 × *g*, 4 °C) to remove cell debris. The supernatant was filtered through a 0.45 µm pore size, hydrophilic PVDF, 47 mm membrane (Merck, HVLP04700) before loading on the respective affinity column.

MBP-16E6_4C4S, MBP-16E6_4C4S-LxxLL(E6AP), and MBP-31E6_2C2S-LxxLL(E6AP) were applied to an MBP affinity column [self-packed amylose column, 30 ml amylose resin (New England Biolabs, #8021) equilibrated with E6-Buffer A [20 mM Tris-HCl, 200 mM NaCl, 3 mM $MgCl_2$, 10 µM $ZnCl_2$, 5 % v/v glycerol, 1 mM TCEP, pH 7.4, at 8 °C]. The unbound proteins were washed away by E6-Buffer A, followed by the wash with E6-Buffer B, a high salt buffer [E6-Buffer A containing 1 M NaCl], to remove nucleic acids and non-specific bound proteins. Proteins were eluted with E6-Buffer C [E6-Buffer A containing 10 mM maltose (Carl Roth, 8951)]. Elution fractions were pooled and centrifuged overnight at 100 000 × *g* to sediment agglomerates. The supernatant was concentrated in a 30 kDa cutoff spin column (Merck, #UFC903008) before being applied to a HiLoad 16/600 Superdex 200 pg (GE Healthcare) pre-equilibrated with SEC buffer [20 mM HEPES, 200 mM NaCl, and 1 mM TCEP, pH 7.5, at 8 °C]. Each time, 4 mL of 15 – 20 mg/mL E6 proteins were loaded. Fractions containing monomeric proteins were pooled (elution volume of ~85.5 mL for MBP-16E6_4C4S-LxxLL and 86.5 mL for MBP-16E6_4C4S), concentrated if needed (>800 µM for fluorescence polarization, >400 µM for analytical ultracentrifugation), flash frozen in liquid nitrogen, and stored at −80 °C.

His_6-TEV-GGG16E7 and His_6-TEV-GGG31E7 were subjected to Ni-NTA affinity column equilibrated with E7-Buffer A [20 mM Tris-HCl, 300 mM NaCl, 3 mM $MgCl_2$, 10 µM $ZnCl_2$, 5 % v/v glycerol, 1 mM TCEP, pH 7.4, at 8 °C]. The unbound proteins were washed away by E7-Buffer A, followed by the wash with high salt buffer E7-Buffer B [E7-Buffer A containing 1 M NaCl] to remove nucleic acids and non-specific bound proteins. Proteins were eluted with E7-Buffer C [E7-Buffer A containing 500 mM imidazole (Carl Roth, X998]. Elution fractions were pooled and centrifuged overnight at 100 000 × *g* to sediment agglomerates. The supernatant was diluted in AEX-Buffer A [20 mM Tris-HCl, 1 mM TCEP, pH 8 at 8 °C] to obtain a concentration of NaCl of less than 20 mM and applied onto an anion exchange column (HiTrap Capto Q 5mL, GE Healthcare) equilibrated with AEX-Buffer A. The unbound proteins were washed away with AEX-Buffer A. The E7 proteins were eluted with AEX-Buffer B [AEX-Buffer A containing 1 M NaCl]. Fractions containing E7 proteins were applied on HiLoad 16/600 Superdex 200 pg equilibrated with SEC buffer for size exclusion chromatography with 5 mL loaded each time. Fractions

containing His$_6$-TEV-GGG-E7 dimers were pooled (elution volume of ~88 mL), and concentrated to 50 μM for His$_6$-TEV tag cleavage.

For control experiments, the protein of MBP alone was purified from *E. coli* biomass (~20 g). The biomass was resuspended in MBP-Buffer A [20 mM Tris-HCl, 200 mM NaCl, 1 mM EDTA, pH 7.4 at 8 °C]. Approximately 8 mg of lysozyme, 1 μL Benzonase® Endonuclease per 2 g wet biomass, and 1 tablet cOmpleteTM EDTA-free Protease Inhibitor Cocktail per 50 mL buffer were added, and incubated with gentle stirring for one hour in the cold room (4 - 8 °C). Then, the cells were lysed as described above. The proteins were applied on MBPTrap HP 5 mL (GE Healthcare, 28918779) that were equilibrated with MBP-Buffer A followed by a wash with high salt buffer MBP-Buffer B (MBP-Buffer A containing 1 M NaCl) to remove nucleic acids and non-specific bound proteins. The proteins were eluted in MBP-Buffer C (MBP-Buffer A containing 10 mM maltose). The elution fractions were applied on HiLoad 16/600 Superdex 200 pg equilibrated with SEC buffer for size exclusion, with 4 mL of proteins loaded each time. Fractions containing MBP monomers were pooled (elution volume of ~88.5 mL), concentrated to >800 μM for fluorescence polarization, flash frozen in liquid nitrogen, and stored at −80 °C.

TEV protease and *Sortase A* were purified as previously described (2, 3).

Cleavage of His$_6$-TEV tag and *Sortase A*-based protein labeling

The purified His$_6$-TEV-GGG16E7 and His$_6$-TEV-GGG31E7 at 50 μM were incubated overnight with the in-house purified TEV protease at a molar ratio of 20:1. After overnight cleavage, the proteins were centrifuged for 1 hour at 100 000 × *g* at 4 °C to sediment aggregates. Then, the TEV protease was separated from the E7 proteins by loading 5 mL supernatant each time on HiLoad 16/600 Superdex 200 pg coupled with 1 mL HisTrap column upstream, equilibrated with SEC buffer, for an online pre-separation of the TEV protease and any non-cleaved E7 proteins containing His$_6$-TEV tag. The cleaved E7 proteins would run through and into HiLoad 16/600 Superdex 200 pg for size exclusion chromatography. Fractions containing GGG-E7 dimers were pooled (elution volume of ~89 mL), concentrated to > 400 μM dimeric concentration for fluorescence polarization, flash frozen in liquid nitrogen, and stored at −80 °C. The cleaved E7 proteins would now

reveal the triple-glycine motif (GGG) at its N-terminus, which is essential for *Sortase A* (*Srt A*). To label the E7 proteins, 25 µM *Sortase A* and 100 µM fluorescein-LPETGGRR were incubated with 50 µM GGG- E7 proteins after adding 10 mM $MgCl_2$ and 5 mM $CaCl_2$ co-factor. The reaction was incubated overnight with gentle stirring in the cold room (4 - 8 °C). After that, the proteins were centrifuged at 100 000 × *g* at 4 °C before applying 5 mL supernatant each time on HiLoad 16/600 Superdex 200 pg coupled with 1 mL HisTrap column upstream, equilibrated with SEC buffer, in which the His_6-*Sortase A* will be trapped in the 1 mL HisTrap column. In contrast, the fluorescein-labelled-E7 (fl-E7) proteins would run through into HiLoad 16/600 Superdex 200 pg for size exclusion chromatography. Fractions containing fl-E7 dimers were pooled (elution volume of ~89 mL), concentrated to 55 µM, flash frozen in liquid nitrogen, and stored at −80 °C.

Synthesis and purification of 16E7CR1/2 (1-44) peptides

The 16E7CR1/2 (1-44) peptide (HGDTPTLHEYMLDLQPETTDLYCYEQLNDSSEEEDEIDGPAGQ) and the corresponding fl-16E7CR1/2 (1-44) peptide were synthesized using standard Fmoc/tBu chemistry on a multiple peptide synthesizer Syro II (MultiSyntechTech, Witten, Germany) on a Rink-Amid resin (4, 5). After removing the N-terminal Fmoc group the resin was divided into two parts. One part was deprotected with reagent K for 3 hours. After precipitation in cold diethylether, the crude peptide was lyophilized.

The residual resin was coupled with 6-Carboxy-fluorescein (6-FAM, Sigma Aldrich) in a 4 molar excess using the TBTU-coupling procedure for 3 hours under light protection. Deprotection was performed according to the procedure for the free peptide. 16E7CR1/2 (1-44) peptides were purified by reversed-phase HPLC on a Reprosil C_{18} column (7 µm; 100 Å; 10x 250 mm; Dr. Maisch, Ammerbuch) using gradient elution with eluent A [0.05 % TFA in water] and eluent B [0.05 % in acetonitrile-water (80:20, v/v %)]. The gradient was from 20 % to 80 % eluent B in 30 minutes at a flow rate of 2.5 ml/min. UV detection was performed at 214 nm. All eluents are degassed under a vacuum to prevent disulfide formation.

Purities were ≥95 % as determined by analytical reversed phase-HPLC. Molecular masses of the labeled and non-labeled 16E7CR1/2 (1-44) were determined by Maldi-TOF-analysis on a Bruker Daltonics (Reflex IV, Germany) giving the correct masses$[M+H]^+$ of 4897.1 Da for the non-labeled 16E7CR1/2 peptide and 5255.3 Da for the fl-16E7CR1/2 peptide.

References

1. Roos, N., Breiner, B., Preuss, L., Lilie, H., Hipp, K., Herrmann, H. *et al.* (2020) Optimized production strategy of the major capsid protein HPV 16L1 non-assembly variant in E. coli Protein Expr Purif **175**, 105690 10.1016/j.pep.2020.105690
2. Tropea, J. E., Cherry, S., andWaugh, D. S. (2009) Expression and purification of soluble His(6)-tagged TEV protease Methods Mol Biol **498**, 297-307 10.1007/978-1-59745-196-3_19
3. Barthels, F., Marincola, G., Marciniak, T., Konhauser, M., Hammerschmidt, S., Bierlmeier, J. *et al.* (2020) Asymmetric Disulfanylbenzamides as Irreversible and Selective Inhibitors of Staphylococcus aureus Sortase A ChemMedChem **15**, 839-850 10.1002/cmdc.201900687
4. Zaidi, N., Herrmann, T., Baechle, D., Schleicher, S., Gogel, J., Driessen, C. *et al.* (2007) A new approach for distinguishing cathepsin E and D activity in antigen-processing organelles FEBS J **274**, 3138-3149 10.1111/j.1742-4658.2007.05846.x
5. Jores, T., Klinger, A., Gross, L. E., Kawano, S., Flinner, N., Duchardt-Ferner, E. *et al.* (2016) Characterization of the targeting signal in mitochondrial beta-barrel proteins Nat Commun **7**, 12036 10.1038/ncomms12036
